# Embryonic Lead Acetate Exposure Induces Seizure-like Activity in Zebrafish Larvae

**DOI:** 10.3390/biomedicines14040897

**Published:** 2026-04-15

**Authors:** Angela Gyamfi, William A. Cisneros, Priyadharshini Manikandan, Christopher A. Subi-Kasozi, Theodore R. Cummins, James A. Marrs

**Affiliations:** Department of Biology, Indiana University Indianapolis, Indianapolis, IN 46202, USA; angyamfi@iu.edu (A.G.); wcisnero@iu.edu (W.A.C.); pmanikan@iu.edu (P.M.); csubikas@iu.edu (C.A.S.-K.); trcummin@iu.edu (T.R.C.)

**Keywords:** zebrafish, lead, seizure, neurotoxicity, neurobehavior

## Abstract

**Background/Objectives**: Despite the decades-old ban on lead in fuel, plumbing, consumer goods, industrial processes, and various materials, it remains a public health threat due to its persistent nature. Zebrafish (*Danio rerio*) are highly effective for modeling several disorders, including those affecting neurological and behavioral functions, and are well-suited for assessing the impact of environmental toxins like lead. This study aimed to investigate the neurodevelopmental effects of embryonic lead exposure using the zebrafish model system. **Methods**: Embryos were exposed to lead acetate (PbAc) at concentrations ranging from 0.3 to 0.7 µg/mL using an exposure window of 6 to 48 h post-fertilization (hpf). **Results**: PbAc exposure produced sublethal teratogenic effects in a subset of larvae across concentrations, including tail and spinal deformities, craniofacial abnormalities, and uninflated swim bladder observed at 7 dpf. At 3 days post-fertilization (dpf), spontaneous circle swimming behavior suspected to be seizure-like was observed in the lead-exposed larvae and was more pronounced under light conditions in a dose-dependent manner. Electrophysiological recordings confirmed that larvae exhibiting circle swimming behavior had heightened neural activity, indicating a potential seizure-like phenotype driven by lead exposure. **Conclusions**: Our findings suggest that embryonic lead exposure leads to morphological defects and seizure susceptibility, demonstrating lead’s neurotoxic potential during early development. Seizure-like behaviors occurred in a non-linear concentration-dependent manner with a photosensitive component, and elevated baseline neural excitability was confirmed by local field potential (LFP) recordings.

## 1. Introduction

Lead’s abundancy, malleability, low electrical conductivity, and resistance to corrosion made it a desirable metal to use in many everyday items such as underground water pipes, household fixtures, children’s toys, paint, and as a gasoline additive [1,2,3]. Despite its many applications, lead is among the most toxic heavy metals found on Earth [2]. Although now banned in everyday goods, use of old consumer products or leaching of lead into water and soil can result in continual exposure and entry into the food chain [4,5]. Lead’s environmental persistence remains a significant concern, as exposure can still occur despite the discontinuation of its use in many consumer products [3]. The United States Centers for Disease Control and Prevention (CDC) identifies lead as harmful at any detectable level as it has no biological role in the body, noting that even blood level concentrations as low as 3.5 μg/dL can contribute to learning and behavioral problems in children [6,7].

Lead exposure can occur through several routes, including ingestion, inhalation, skin contact, and transplacental transmission [8]. After initial exposure, blood lead levels (BLLs) rise rapidly, then gradually redistribute into other compartments of the body [1]. Once in the body, about 5–15% is excreted, while the remainder can accumulate in soft tissues and bones, where it may persist for 10 to 30 years [9]. In pregnant women, the increased physiological demand for calcium leads to the mobilization of lead from bone stores back into the blood stream where it can cross the placental barrier and affect fetal development [8,10]. The clinical presentations of gestational lead poisoning include premature birth, mild mental retardation, encephalopathy, headache, renal failure, convulsions, behavioral disorders, and reduced IQ [8,11]. As a potent neurotoxin, its effects on the developing brain are enormously damaging, putting fetal and child development at greater risk than adult exposure [8,12,13].

Studies on mammalian models, including rats and mice, have linked lead exposure to severe outcomes, such as renal cancer, glioma, and convulsions, underscoring the cross-species health risks posed by lead toxicity [14,15]. Similarly, studies using zebrafish have associated lead exposure to morphological, behavioral, learning, memory, and sensorimotor deficits [12]. Zebrafish serve as an effective model for studying lead toxicity and seizure due to their genetic similarity to humans (70% sequence homology) and sensitivity to neurotoxicants that parallel human developmental effects [4]. Their rapid development, transparent embryos, and characterized seizure-like behavioral responses, which can be easily quantified and correlated with neural activity, make them ideal for investigating the behavioral effects of heavy metal exposure, such as lead-induced neurotoxicity and seizure mechanisms [12,16].

Behaviorally, seizures in zebrafish are characterized by repetitive rapid swimming, freezing, and loss of posture or toppling over [17,18]. Seizure behavior in zebrafish larvae can be identified through a combination of behavioral and functional assays, such as calcium imaging and electrophysiology [19,20]. Zebrafish offer several advantages for investigating neurodevelopmental processes [21]. Notably, zebrafish embryos develop externally and remain transparent during early stages, facilitating in vivo, whole-brain analysis without the need for invasive procedures [12,22,23].

Calcium plays a critical role in many cellular processes, including neurotransmitter release and synaptic signaling, making it a reliable proxy for monitoring neural activity [24,25,26]. Calcium imaging using genetically encoded calcium indicators leverages the transparent nature of the larvae allowing researchers to visualize brain activity in vivo [25]. The biochemical disruptions caused by lead can be attributed to its ability to substitute for calcium ions and alter calcium signaling [13]. This substitution leads to alterations in neurotransmitter release, neuronal excitability, and cellular homeostasis [12]. At the molecular level, lead can interfere with the regulatory functions of calcium through ionic mimicry where it displaces calcium from its active sites and through opportunistic binding at non-canonical binding sites [27,28]. Lead can mimic calcium and bind to calmodulin (CaM) under low calcium conditions due to its similar coordination geometry [27,28]. CaM exhibits a 3-to-8-fold higher affinity for lead than for calcium and binding to the non-canonical or secondary sites can induce conformational changes that impair its normal function and disruption of downstream calcium signaling pathways [28]. These disruptions can alter neural activity patterns, which can be captured using electrophysiology. Electrophysiological recordings, which detect patterns of brain activity, offer a direct insight into seizure-related neural dysfunction and complement calcium imaging by enabling the spatial and temporal characterization of seizures [29].

To choose a lead concentration that was environmentally relevant and that would allow for morphological, behavioral, and molecular changes, zebrafish embryos were exposed to varying amounts of lead in their environment to characterize the general response of the embryos treated with a range of 0.3–0.7 μg/mL (300–700 ppb). Lead exposure is known to be harmful even at low concentrations. Although lead poisoning is often asymptomatic, elevated blood levels ≥ 45 μg/dL (450 ppb) can cause nonspecific symptoms such as headache, abdominal pain, anorexia, and constipation, while more severe neurological effects, including clumsiness, agitation, stupor, drowsiness, vomiting, and convulsions, may occur at higher levels [7]. Importantly, even BLLs below 5 μg/dL (50 ppb) in children, who may appear asymptomatic, have been consistently linked to irreversible deficits in neurocognitive and behavioral development [30,31,32].

The American Academy of Family Physicians (AAFP) reported on screening and management based on previous CDC recommendations from 2018; children with confirmed BLLs below 5 μg/dL (50 ppb) need only routine developmental and nutritional assessments, education about lead sources, and regular age-appropriate screening [30]. As levels rise, interventions progress from nutrition counseling and environmental investigation (≥5 μg/dL (50 ppb)) to neurodevelopmental evaluation, laboratory testing, and lead hazard reduction (≥20 μg/dL (200 ppb)), with chelation therapy and possible hospitalization for levels ≥45 μg/dL (450 ppb), and mandatory hospitalization and specialist-guided chelation for levels ≥70 μg/dL (700 ppb) [30]. The range of 0.3–0.7 μg/mL (300–700 ppb) was chosen as it corresponds to BLLs at which medical monitoring and intervention are recommended, indicating that these concentrations are environmentally relevant and represent realistic exposure scenarios associated with adverse health outcomes.

Zebrafish embryos were treated with lead for a time range of 6–48 h post-fertilization (hpf) as this model allows the investigation of how environmentally levels of lead exposure may contribute to long-term neurodevelopmental effects that are not immediately observable during early development. Studies on zebrafish using ≥5000 ppb recorded a significantly high mortality rate at ≤100 ppb showing no observable morphological alterations as compared to the controls and reported altered expression in critical genes of the central nervous system (CNS) even at this low concentration, especially at 72 hpf [33]. The range of PbAc concentrations (0.3–0.7 μg/mL) used in this study was chosen to capture subtle neurodevelopmental changes without inducing extreme toxicity, based on Peterson and colleagues’ [33] findings that intermediate treatment levels (500 ppb) did not elicit particularly severe effects, while still reflecting concentrations above the current safe limit for children and within the range of concerning lead exposure levels in the US.

Reports from the National Resources Defense Council (NRDC) indicate that 0 ppb is the only safe level of lead exposure in tap water; however, between 2021 and 2024, approximately 81% of the US population using community water systems had water containing at least 1 ppb of lead, 36% had 5 ppb or more, 14% had 10 ppb or more, and 4% had 15 ppb or higher [34]. While these levels are substantially lower than the concentrations used in zebrafish studies, higher doses are necessary due to the difficulty in directly correlating zebrafish exposure concentrations with human BLLs. The complex binding dynamics and absorption properties of lead make it challenging to approximate a zebrafish developmental dose equivalent to that of a child [33]. Additionally, because lead has a biological half-life of about 35 days and can accumulate in bones for decades [9], higher laboratory concentrations help to model the cumulative and body burden of lead. This includes maternal stores that can be mobilized and transferred to the developing embryo through the placenta, mimicking the exposure risk faced by babies during early development. Although community water system lead levels are lower, the extent to which maternal lead burdens are passed to offspring and their impact on neurodevelopment remain unclear, and the range chosen for this study falls within ranges considered concerning for human health. This study remains relevant because lead continues to leach into drinking water across the US, exposing millions of people to levels that are associated with adverse developmental and neurological effects. However, no studies to date have established a link between embryonic lead exposure and the induction of seizures in zebrafish. To address this gap, zebrafish were used to investigate the neurodevelopmental effects of embryonic lead acetate (PbAc) exposure. It was hypothesized that developmental lead exposure would disrupt CNS function by inducing teratogenic effects and neuronal hyperactivity in the larval zebrafish.

## 2. Materials and Methods

### 2.1. Zebrafish Husbandry

The Indiana University Policy on Animal Care and Use guidelines were followed, and all experiments were approved (protocol number: SC361R; approval date: 20 October 2025) by the Indiana University Indianapolis School of Science Institutional Animal Care and Use Committee (IACUC). Adult zebrafish (AB strain approximately 1 year old) were raised and maintained under standard laboratory conditions [35], in an Aquaneering water housing standalone system (San Diego, CA, USA).

### 2.2. Breeding and Egg Production

Adult zebrafish were isolated into a spawning tank overnight in a 1:1 or 2:1 male/female ratio. Embryos were collected and washed with embryo medium (EM) (1.5 g instant ocean salt in 4 L deionized water). Embryos were incubated at 28.5 °C and raised on a 14/10 h light/dark cycle after 2 days post-fertilization (dpf), permitting normal development.

### 2.3. Lead Acetate Preparation and Exposure of Embryos

Healthy embryos with normal blastula formation at 6 hpf were evenly distributed into 6-well plates with a range of around 25–30 embryos per well at random into control or treatment groups. Control embryos were washed with fresh embryo medium or lead treatment media based on the experimental parameters daily.

A 1 g/L lead acetate trihydrate (PbAc; purity 99%, St. Louis, MO, USA, Sigma) stock was made in distilled water. Experimental stocks of 0.3 µg/mL, 0.4 µg/mL, 0.5 µg/mL, 0.6 µg/mL, and 0.7 µg/mL PbAc were diluted in embryo medium; it is noted that these represent nominal concentrations as the partial precipitation of PbCl_2_ may occur upon dilution in NaCl containing embryo media. In all assays performed, PbAc treatment occurred at a range of 6–48 hpf. This window was chosen to span zebrafish development from gastrulation to prehatching (end of embryogenesis), which are critical stages of neurodevelopment, including active neurogenesis [36]. Following the 6–48 hpf range exposure period, the PbAc solutions were replaced with fresh embryo medium daily until the experiments were completed (3 or 7 dpf). Each assay was an independent experiment (larvae were not reused).

### 2.4. Teratogenic Effects Assessment

Teratogenic effects were assessed to determine a suitable concentration range for further analysis. A range of 25–30 larvae was monitored daily until 7 days, and mortality rates were recorded to compare treated groups with untreated groups. A Leica (model MZ12) dissecting microscope with camera (Leica DFC 450 C, Wetzlar, Germany) was used to image 7 dpf live larvae. Larvae were embedded in 3% methyl cellulose to reduce motility, and lateral and dorsal images were taken for each treatment group. Teratogenic effects evaluated were spinal and tail deformation, swim bladder inflation, and craniofacial abnormalities. Three representative larvae from control (unexposed larvae) and three representative larvae from each treatment group showing lead-induced abnormalities were imaged in both dorsal and lateral views.

### 2.5. Circle Swimming Assay

**Figure 1:**
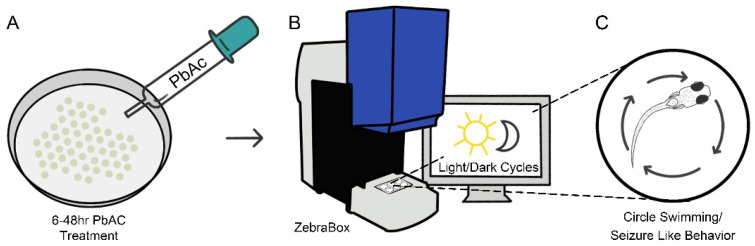
Circle swimming assay to assess seizure-like behavior in lead-exposed 3 dpf zebrafish larvae. (**A**) Zebrafish embryos were treated with PbAc at a range of 6–48 hpf. (**B**) Larvae were placed in a ZebraBox system, acclimatized, and subjected to alternating light/dark cycles. (**C**) Circle swimming behavior defined as a complete 360° turn was recorded and quantified as an indicator of seizure-like activity.

At 3 dpf, spontaneous seizure-like activities were observed in lead-exposed larvae, and behavioral analyses were conducted to quantify the severity and frequency of the phenotype. Three biological replicates were performed. Embryos were transferred into six-well plates, with a range of 25–30 embryos per well and grouped according to varying PbAc concentrations (0.3–0.7 μg/mL). Plates were placed in a ZebraBox at room temperature (ViewPoint Behavior Technologies, Lyon, France). The tracking system was not used due to the small size of 3 dpf larvae. An infrared camera allowed the behavioral assessment in dark (0% illumination) and light (100% illumination) conditions (Figure 1). Parameters for circle swimming behavior were set up for a total of 2.5 h of alternating light and dark cycles. The first 30 min of darkness was designated as an acclimatization period followed by six cycles of 10 min light and 10 min dark for the remaining 2 h. Video recordings were collected and analyzed using video playback software. Videos were reviewed manually and the number of circle swimming events (defined as a complete 360° turn) was recorded (see Video S1).

### 2.6. In Vivo Electrophysiological Recordings in the Optic Tectum of 3 dpf Zebrafish Larvae

**Figure 2:**
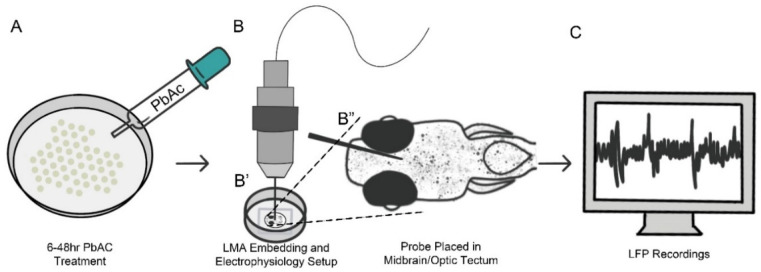
Electrophysiology setup of live zebrafish brain recordings. (**A**) Pretreatment of embryos. (**B**) LFP recording from the optic tectum using a glass microelectrode filled with 2 mM NaCl and measured in the current clamp mode. (**C**) Seizure-like activity assessed by analyzing the frequency and amplitude of paroxysmal spiking events.

To evaluate potential seizure-like effects in 3 dpf larvae showing circle swimming behavior, local field potential (LFP) recordings were carried out to assess brain activity patterns using the method described by Liu and Baraban [29] (Figure 2). Untreated (control) 3 dpf larvae of the AB strain and those pre-treated with 0.4 µg/mL PbAc at a range of 6–48 hpf were embedded in 1% low melt agarose in a 35 mm glass-bottom dish. After allowing the agarose to set, the dish was filled with 2 mL of EM (Figure 2B’). A glass electrode filled with 2 mM NaCl was placed into the optic tectum and recordings were performed in the current clamp mode, low-pass filtered at 1 kHz, high-pass filtered at 0.1 Hz, with a digital gain of 10 and sampling interval of 10 µs (EPC10, Heka Electronic, Lambrecht, Germany) (Figure 2B”). The larvae were allowed to habituate with an electrode in place for about 3 min. Then, a 2 min equilibration reading was carried out followed by a baseline recording for 5 min. Following this, 2 mL of a 20 mM pentylenetetrazol (PTZ) solution was added to the 2 mL embryo medium already present in the dish, resulting in a 1:1 dilution and a final PTZ concentration of 10 mM. PTZ is a GABA-A receptor antagonist that reduces inhibitory neurotransmission by blocking chloride ion influx, thereby lowering the seizure threshold and inducing seizure-like activity. It is widely used as a proconvulsant agent in zebrafish and rodent seizure models to assess neural hypersensitivity. The recordings started exactly after the addition of the proconvulsant solution and were continued for 10 min. The EEG treatment was not blinded and recordings from nine larvae were taken per experimental condition. Seizure activity was analyzed according to the amount of spiking paroxysmal events or LFP recorded (Figure 2C).

### 2.7. Statistical Analysis

All statistical analyses were conducted using GraphPad Prism (Prism 10 version 10.2.3) to assess the effects of lead acetate exposure in experimental treatments on zebrafish behavior. Data were first tested for normality using the Shapiro–Wilk test to determine the appropriate statistical approach. For normally distributed data with more than two groups, a Two-Way Repeated Measures (RM) ANOVA was used followed by Tukey’s multiple comparison test for both the circle swimming assay data and electrophysiology recordings. All data are expressed as the mean ± standard error of the mean (SEM), and statistical significance was set at *p* ≤ 0.05.

## 3. Results

### 3.1. Lead-Induced Morphological Alterations in 7 dpf Zebrafish Larvae

**Figure 3:**
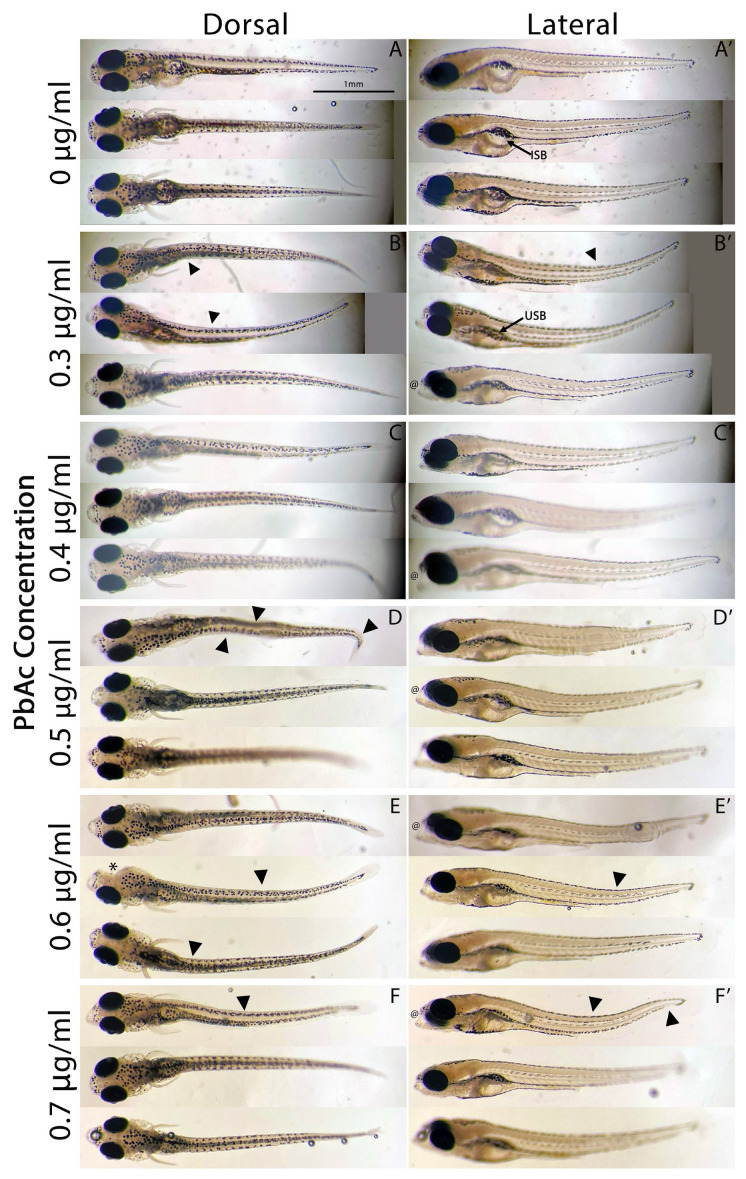
Observed teratogenic effects of PbAc-exposed zebrafish larvae at 7 dpf. Larvae are displayed in the dorsal and lateral orientations: (**A**) control (0 μg/mL) larvae in the dorsal view, (**A’**) control larvae in the lateral view, (**B**–**F**) 0.3–0.7 μg/mL PbAc-treated larvae in the dorsal view, respectively, and (**B’**–**F’**) 0.3–0.7 μg/mL PbAc-treated larvae in the lateral view, respectively. Black arrowheads indicate spinal and tail deformities and @ indicates craniofacial deformities, both visible teratogenic effects of PbAc exposure. ISB—inflated swim bladder; USB—uninflated swim bladder; *—eye loss due to mechanical manipulation during imaging.

Zebrafish embryos were treated with 0.3–0.7 μg/mL range of PbAc from the onset of gastrulation at 6 hpf through the end of organogenesis at 48 hpf because this period encompasses the critical stages of early neurodevelopment, allowing the investigation of how environmentally relevant lead exposure may produce long-term neurodevelopmental effects that may not be immediately apparent during development (Figure 3). For both the treatment of 0.4 μg/mL PbAc and control groups, the observed mortality rate at 3 dpf was zero and one lead-treated larvae died at 4 dpf (n = 40 per group). Morphological abnormalities were observed at 7 dpf across treatment concentrations, though these were not quantified (Figure 3). Malformations observed included spinal, tail and craniofacial deformities, and uninflated swim bladder observed in a subset of larvae across concentrations (Figure 3). Importantly, no extreme defects or lethality were observed at any concentration tested, indicating that the model produced sublethal effects within the range examined (Figure 3D–F,D’–F’).

### 3.2. Lead-Induced Spontaneous Circle Swimming/Seizure-like Behavior

**Figure 4:**
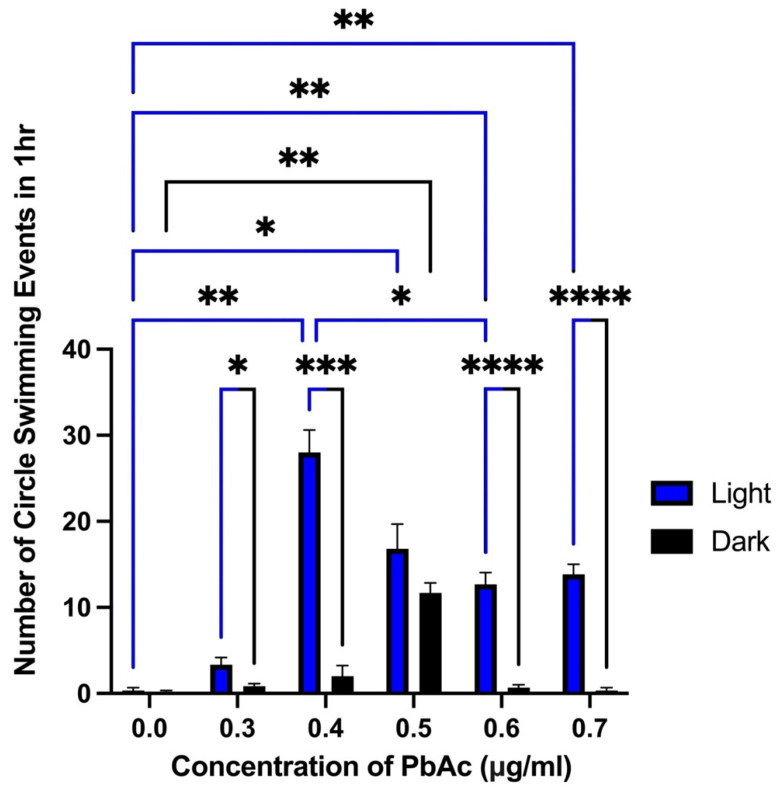
Frequency of circle swimming activity across varying PbAc concentrations in 3 dpf larvae. Bar graph illustrating the frequency of circle swimming behavior in zebrafish larvae exposed to different concentrations of PbAc (0.3–0.7 μg/mL) compared to the control (0.0 μg/mL). Statistical analysis was conducted using Two-Way RM ANOVA followed by Tukey’s post hoc test. Asterisks (*) denote statistically significant differences at *p* ≤ 0.05, n = 25–30 per treatment group; * *p* ≤ 0.0171, ** *p* ≤ 0.0029, *** *p* ≤ 0.0002, and **** *p* ≤ 0.0001.

At 3 dpf, spontaneous circle swimming behavior was observed in lead-exposed groups and characterized using alternating light and dark cycles as previously described. Circle swimming behavior was defined as larvae completing a full 360° turn in approximately 3 s. This behavior was observed at all treated lead concentrations, with the highest frequency occurring in the 0.4 µg/mL PbAc group (Figure 4; Video S1). Notably, across all lead-exposed groups, circle swimming behavior was significantly higher during light cycles than during dark cycles, suggesting possible photosensitivity. It is likely that spinal and tail deformities in the lead-treated larvae influence circle swimming behavior. The ZebraBox tracking system does not identify larvae reliably when they are small. Hyperactivity and circle swimming peaked at the 3 dpf stage. The morphologically affected fish also tend to show increased hyperactivity, increasing in the circle swimming behavior. There are many larvae with less teratogenic effects that also swim in circles. Hyperactivity and circle swimming were generally not observed in control fish. We suspect that the circle swimming measurements underrepresent the total seizure-like events.

### 3.3. In Vivo LFP Recordings from the Optic Tectum of 3 dpf Zebrafish Larvae

**Figure 5:**
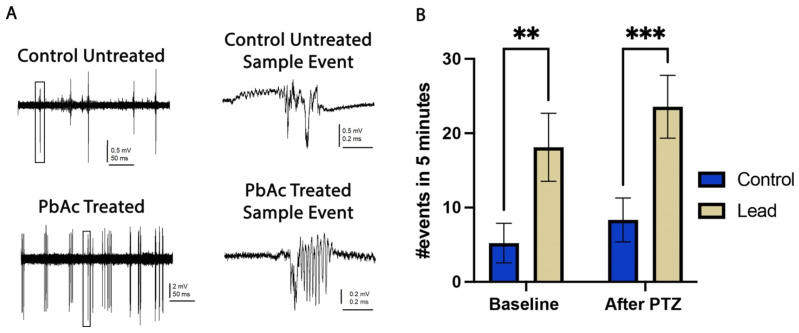
Local field potentials in 3 dpf zebrafish larvae. (**A**) Representative tracings recorded in the presence of EM (top) for untreated and (bottom) lead-treated groups. (**B**) Representative LFP differences/events in 5 min are displayed on the y-axis. The respective treatment groups for pretreatment (control/treated) and acute exposure (baseline and PTZ) are shown on the x-axis. Statistical analysis determined by Two-Way RM ANOVA followed by Tukey’s post hoc test; data are expressed as mean and SEM; statistical significance set at *p*≤ 0.05 and by (n = 9 per treatment group; two biological replicates); ** *p* ≤ 0.0011 and *** *p* ≤ 0.0004.

To obtain a direct measurement of brain activity with high temporal resolution in 3 dpf larvae and to examine whether increased brain activity correlates with circle swimming behavior, we performed LFP recordings (Figure 5). Measurements were performed in the optic tectum, which allowed us to target a brain region highly vulnerable to neurotoxic insults and that could be consistently visualized and probed across all groups, thereby reducing variability between individual zebrafish. Lead-exposed larvae showed significantly higher LFP activity compared to the controls at baseline, demonstrating heightened neural excitability (Figure 5A,B). Following acute exposure to the proconvulsant drug, PTZ, a trend toward increased LFP activity was observed in both control and lead-exposed groups, although not statistically significant (Figure 5B).

## 4. Discussion

Lead exposure remains a significant public health concern, as lead continues to leach into drinking water across the US; while many Americans are in contact with lead concentrations below regulatory levels, chronic exposure to these levels can contribute to BLLs associated with adverse neurodevelopmental outcomes [34]. The 0.3–0.7 μg/mL (300–700 ppb) PbAc range used in this study represents environmentally relevant levels that allow the investigation of morphological and behavioral alterations in zebrafish embryos. This approach helps bridge the gap between environmental lead levels and their potential long-term impacts on neurodevelopment.

The range of concentrations used in this study permitted the observation of morphological alterations across concentrations in a subset of larvae without apparent differences in mortality rates between treated and control groups. Observed abnormalities included spinal and tail deformations, uninflated swim bladder, and craniofacial abnormalities. All experimental groups exhibited less than 10% lethality, indicating that the model does not induce extreme lethality. These findings highlight the teratogenic effects of lead, demonstrating its ability to disrupt normal embryonic development, similar to observations made by Komoike and Matsuoka [15], using a concentration of 100 ppb at a range of 6–72 hpf. Our exposure window in the range of 6–48 hpf spanned zebrafish gastrulation to the pre-hatching stage, critical periods of early development.

One of the principal findings of this study was the observation of seizure-like behaviors at 3 dpf. The observed behavioral phenotype consisted of distinct circular (looping) swimming motions, often followed by episodes of toppling over and brief freezing. Notably, circle swimming behaviors were exacerbated in the presence of light (Figure 2), consistent with a photosensitive component, though this requires further investigation. Light flashes can provoke photosensitive seizures triggered by visual stimulation [37]. The seizure-like activity peaked at 0.4 µg/mL PbAc, with reduced intensity at higher concentrations, suggesting a non-linear, concentration-dependent response. At 0.5 µg/mL, the circle swimming behavior between the light and dark conditions was not statistically significant, suggesting a possible loss of the light/dark effect at that concentration. Higher concentrations (0.6 µg/mL and 0.7 µg/mL), however, retained this effect, although with relatively reduced circle swimming behavior overall. This reduction may reflect emerging paralysis or extensive motor/central nervous system deficits, which may be a subject of future studies. Seizures are a well-established outcome of lead neurotoxicity in both humans and animal models such as rodents [38,39,40].

In zebrafish, the blood–brain barrier (BBB) is immature at 3 dpf and not fully functionally developed, allowing environmental toxins like lead to permeate the brain more readily through immature tight junctions [41,42,43]. This developmental vulnerability may explain why seizure-like behaviors were most evident at this stage. Although BBB disruption has been proposed as a mechanism underlying lead-induced seizures [44,45,46,47,48], previous studies and our findings suggest that lead-induced seizures may occur independently of overt BBB breakdown, such as hemorrhage or edema [39,46]. Seizure behavior observed in this study was more apparent at 3 dpf, where no visible structural abnormalities such as edema or hemorrhage were detected [44,48]. Lead-exposed larvae were comparable to controls at this stage. This supports the hypothesis that lead may directly alter neural excitability, possibly through the disruption of inhibitory neurotransmission or calcium homeostasis [49], rather than acting solely through BBB compromise.

To better understand the neural basis of the seizure-like behaviors observed, we used a GCaMP-expressing transgenic zebrafish line, *Tg(elavl3:soma-GCaMP7f)*, to perform functional calcium imaging and assess real-time neuronal activity in the lead-exposed larvae [50] (see Supplementary Materials). In this line, the elavl3 promoter, a well-characterized pan-neuronal promoter, drives the expression of GCaMP7f, a genetically encoded calcium indicator that increases fluorescence in response to calcium binding [50]. The fluorescence signal was localized to neuronal cell bodies (soma), allowing the precise visualization of calcium activity within individual neurons [50]. This enabled the non-invasive monitoring of neuronal excitability in live, intact larvae with high spatial and temporal resolutions. Using this tool coupled with confocal imaging, we observed considerable variability in fluorescence intensity among individual fish within each treatment group (Figure S2). Although the representative images show a spatial increase in fluorescence intensity in lead-exposed larvae, some individuals exhibited lower fluorescence than controls, even after PTZ exposure (a seizure inducing drug that increases neuronal activity). These differences suggest that lead’s impact on neuronal activity is not uniform and may be influenced by individual biological or experimental variability.

A possible explanation for this variability lies in the differences in lead bioaccumulation and intracellular binding dynamics. At 3 dpf, although there was no free lead in the media, residual lead likely persisted within tissues due to prior uptake. This retained lead may interact with intracellular targets such as calmodulin (CaM), a critical calcium-binding protein involved in regulating neuronal signaling. At lower concentrations, lead can mimic calcium by binding to CaM with even greater affinity, transiently activating it and potentially enhancing calcium signaling [28]. In contrast, at higher concentrations, lead may interact with non-canonical or secondary sites on CaM, leading to structural distortion and impaired function [28]. These concentration-dependent interactions, activation at low levels and inactivation at high levels [28], highlight the complex dynamics of lead neurotoxicity and may underlie the seizure-like behaviors observed in the lead-exposed larvae. Therefore, the variability in fluorescence intensity among individual fish may reflect differences in the extent of residual lead binding and its effects on intracellular calcium signaling. Although calcium biosensor assays, such as those using the GCaMP line, are powerful tools for indirectly measuring neuronal activity, their interpretation in the context of inorganic lead exposure is more complex. Therefore, using GCaMP may not provide an accurate representation of neuronal activity in lead-exposed systems, and direct electrophysiological measurements were warranted.

We therefore performed direct measurements of brain electrical activity using electrophysiology to assess changes in neural function associated with lead-exposed larvae at 3 dpf. Specifically, we employed LFP recordings, which capture the collective electrical activity from small populations of neurons within a brain region [51]. This technique is particularly well-suited for zebrafish as behaviors can be quantified, offering a direct measure of neural activity in real time [29]. In the absence of the acute seizure-inducing agent (PTZ) used in this study, lead-pretreated zebrafish larvae exhibited more frequent ictal-like events and greater firing rates compared to untreated controls. This suggests that developmental lead exposure primes neural circuits, resulting in elevated baseline excitability. The optic tectum, being a major visual processing center, plays a critical role in sensorimotor behavior and photosensitivity, and is highly sensitive to neurotoxic insults [52,53]. The altered excitability observed in this brain region as the principal site of LFP recordings in this study further supports lead’s neurotoxic potential.

The spontaneous increase in neuronal firing observed in lead-exposed larvae provides functional evidence that early-life lead exposure disrupts normal network homeostasis. Following PTZ administration, a trend toward more pronounced and frequent neural events was observed in both control and lead-exposed groups, although this did not reach statistical significance in either group. These findings suggest that the elevated baseline neural excitability induced by developmental lead exposure may engage similar pathways to those targeted by PTZ, though this warrants further investigation. It is possible that a larger sample size or higher PTZ concentrations might reveal more robust effects. These results align with previous findings, supporting the idea that lead affects both spontaneous activity and responses to proconvulsant stimuli [44,54]. The presence of ictal-like discharges even under baseline conditions without an acute trigger suggests that developmental lead exposure may promote the formation of hyperactive neurons.

Another possible mechanism for the heightened excitability observed is the disruption of inhibitory neurotransmitter systems, such as GABA [40,45,55]. Although our study did not directly measure GABA levels, previous rodent studies have found region-specific reductions in GABA, particularly in the cerebellum, even when whole-brain levels remain unchanged [40,55]. Lead may interfere with GABA synthesis or function by altering the activity of key enzymes such as glutamic acid decarboxylase (GAD) or GABA transaminase (GABA-T), or by disrupting receptor-mediated signaling [40,45]. Additionally, lead may impair glycinergic pathways, further destabilizing excitatory–inhibitory balance. Such GABAergic disruption may contribute to the elevated baseline neural excitability and ictal-like discharges observed in our lead-exposed larvae. Developmental lead exposure has also been reported to alter NMDA receptor composition and excitatory signaling dynamics in mammalian systems [56]. Zebrafish possess homologous NMDA receptor subunits [57], and the ictal-like discharges observed under baseline conditions in our lead-exposed larvae may reflect similar disruptions to excitatory signaling dynamics. Lead has also been reported to act as a potent non-competitive antagonist of NDMA receptors, altering their structure and function [56,58]. Impairment of downstream NMDAR signaling pathways may further contribute to the synaptic dysregulation underlying the hyperexcitability observed in our model [59,60,61]. We therefore propose that the binding dynamics of lead on key ion channels, calcium binding proteins, and excitatory/inhibitory receptors predispose neural circuits to hyperexcitability, as evidenced by the elevated baseline LFP activity and seizure-like behaviors observed in our study.

## 5. Conclusions

Consistent lead exposure during early development poses significant risks to growth, neurodevelopment, and overall health. These effects may not be immediately apparent but can manifest later in life as impairments in learning, communication, and behavior. In this study, we used zebrafish embryos to investigate the neurodevelopmental impact of embryonic PbAc exposure. Our findings, based on behavioral and electrophysiological analyses, demonstrate that embryonic lead exposure induces seizure-like behaviors in a non-linear, concentration-dependent manner with a photosensitive component, and elevates baseline neural excitability as confirmed by LFP recordings. These results suggest that lead exposure primes neural circuits toward hypersensitivity, potentially through the disruption of GABAergic inhibition and NMDA receptor-mediated signaling. Overall, these findings show lead’s teratogenic and neurotoxic potential during early development. Future work should examine neurochemical changes and detailed neural circuit defects to fully capture the complex impacts of developmental lead exposure on neural function and behavior.

## Data Availability

The original contributions presented in this study are included in the article/Supplementary Material. Further inquiries can be directed to the corresponding author.

## Supplementary Materials

The following supporting information can be downloaded at: https://www.mdpi.com/article/10.3390/biomedicines14040897/s1, Figure S1: Calcium-dependent fluorescence changes in 3 dpf zebrafish larvae using GCaMP imaging; Figure S2: GCaMP-based calcium imaging in 3 dpf zebrafish larvae under baseline and PTZ conditions; Video S1: ZebraBox video imaging of 3 dpf zebrafish larvae.
