# Supplementary material for: Embryonic Lead Acetate Exposure Induces Seizure-like Activity in Zebrafish Larvae
Source: Biomedicines. 2026 Apr 15;14(4):897. doi: 10.3390/biomedicines14040897 (PMC13112978; doi:10.3390/biomedicines14040897)
Supplement: Supplementary file 1 [file biomedicines-14-00897-s001.zip › biomedicines-4194302-supplementary.pdf]

Supplement

Supplementary Methods: Zebrafish Husbandry The Indiana University Policy on Animal Care and Use guidelines were followed, and all experiments were approved by the Indiana University Indianapolis School of Science Institutional Animal Care and Use Committee (IACUC). Adult zebrafish (Tg(elavl3:soma-GCaMP7f) transgenic line; approximately 1 year old) were raised and maintained under standard laboratory conditions (Westerfield, 2000), in an Aquaneering water housing standalone system (San Diego, CA).

GCaMP Calcium Biosensor Assay

To analyze potential seizure-like activity at 3 dpf (the timepoint when circle swimming behavior emerges and is most pronounced), calcium imaging of neuronal activity was performed using a genetically encoded calcium indicator (Figure S1). The transgenic Tg(elavl3:soma-GCaMP7f) line (calcium indicator) used in this study was obtained from the Synthetic Neurobiology Group at the MIT Media Laboratory, Massachusetts Institute of Technology (MIT), Cambridge, MA, USA (Shemesh et al., 2020). As anesthetics modify neuronal activity, experiments were done using agarose-embedded larval zebrafish to reduce movement. Larvae were embedded in 1% low melt agarose in a 35 mm glass bottom dish. Confocal images were obtained using a Zeiss LSM 700 laser-scanning confocal imaging system. The eGFP laser light was used to excite the GCaMP fish at a wavelength of 488 nm. Emission filters were applied for a Z-stack series of 12 slices for a total of 33 µm using a 20x objective. Control groups and 0.4 µg/mL PbAc treated groups of 3 dpf larvae were selected for this analysis. Images were taken of the optic tectum/midbrain, hindbrain, and cerebellum in a dorsal orientation and classified as baseline images. Without disturbing the agar imbedded larvae, 2 mL of 10 mM PTZ was added to the dish, incubated for 20 minutes before taking the images using the same parameters as the baseline. Fluorescence in the midbrain, cerebellum, and hindbrain was measured in ImageJ. The initial baseline fluorescence in the control group was normalized to 1. Fluorescence values from the acute exposure images were divided by the baseline values to obtain normalized fluorescence ratios. This procedure was followed for all lead-treated groups (n=8 per group). The average fluorescence intensities of the lead-treated groups were then compared to those of the controls.

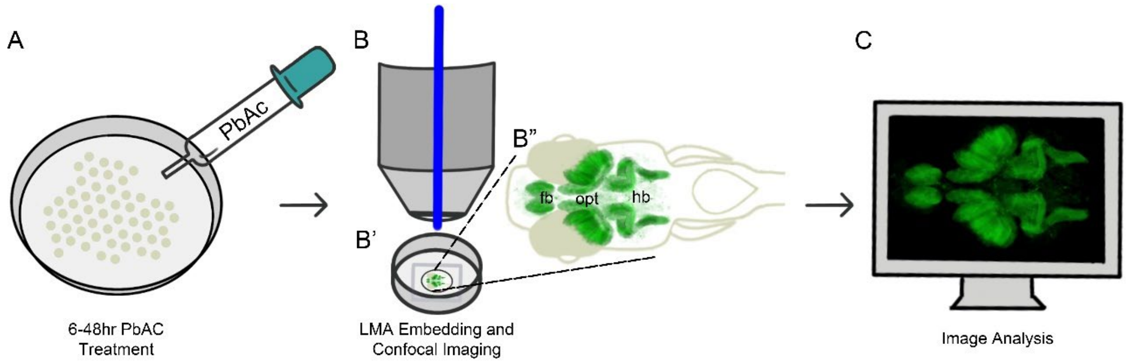

Figure S1: Calcium dependent fluorescence changes in 3 dpf zebrafish larvae using GCaMP imaging. (A) Pretreatment of embryos. (B) Embedding of larvae in low melt agar and confocal imaging setup using the Tg(elav3:soma-GCaMP7f) line with 488 nm excitation and Z-stack acquisition on Zeiss LSM 700. (C) Image analysis of fluorescence intensity changes ($\Delta F/F_0$) in defined brain regions representing calcium dynamics between control and lead treated groups and before and after PTZ exposure.

Supplementary Results: GCaMP Calcium Biosensor Imaging

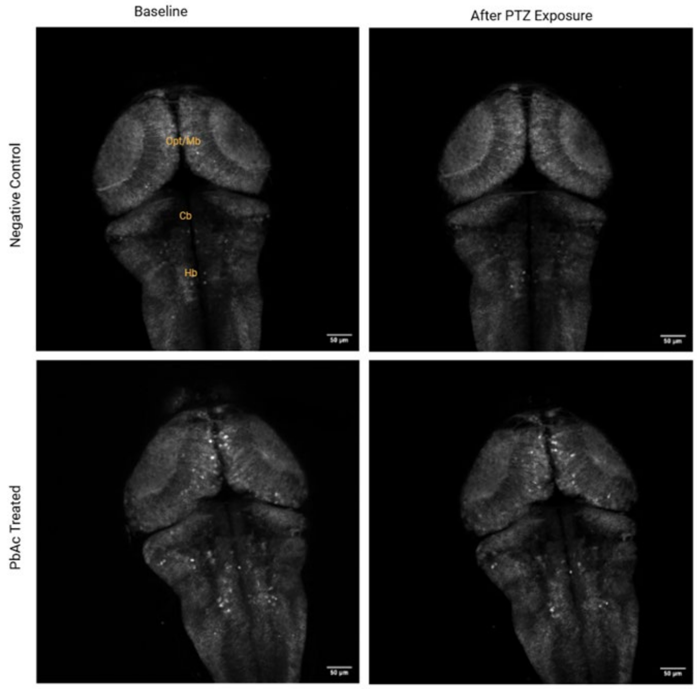

Figure S2: GCaMP-based calcium imaging in 3 dpf zebrafish larvae under baseline and PTZ conditions. Representative dorsal view of Z-stack images (16 optical slices) from Tg(elavl3:soma-GCaMP7f) transgenic zebrafish, visualizing calcium-dependent fluorescence in the brain. Panels include control larvae (top left: baseline; top right: post-

PTZ) and PbAc-exposed larvae (bottom left: baseline; bottom right: post-PTZ). Imaging was performed at 3 dpf, capturing key brain regions including the optic tectum/midbrain (Opt/Mb), cerebellum (Cb), and hindbrain (Hb). Fluorescence seen as bright spots in the neuronal soma in each brain region reflects calcium activity/neural excitation (n=8 per group).

Video S1: ZebraBox video imaging of 3 dpf zebrafish larvae. The control (left well) and 0.5 μg/mL PbAc (right well) treatment groups are shown in the dark for 30 seconds, followed by 90 seconds in the light. Hyperactivity is evident. Circle swimming events are frequently observed in the PbAc treatment group.
